# High nuclear expression of proteasome activator complex subunit 1 predicts poor survival in soft tissue leiomyosarcomas

**DOI:** 10.1186/s13569-016-0057-z

**Published:** 2016-10-01

**Authors:** Sha Lou, Arjen H. G. Cleven, Benjamin Balluff, Marieke de Graaff, Marie Kostine, Inge Briaire-de Bruijn, Liam A. McDonnell, Judith V. M. G. Bovée

**Affiliations:** 1 Center for Proteomics and Metabolomics, Leiden University Medical Center, Albinusdreef 2, Postzone L1-Q, Postbus 9600, 2300 RC Leiden, The Netherlands; 2 Department of Pathology, Leiden University Medical Center, Leiden, The Netherlands; 3 Maastricht MultiModal Molecular Imaging Institute (M4I), Maastricht University, Maastricht, The Netherlands; 4 Fondazione Pisana per la Scienza ONLUS, Pisa, Italy

**Keywords:** Proteasome activator complex subunit 1, Prognostic biomarker, Sarcoma, Leiomyosarcoma, Soft tissue sarcoma, Immunohistochemistry

## Abstract

**Background:**

Previous studies on high grade sarcomas using mass spectrometry imaging showed proteasome activator complex subunit 1 (PSME1) to be associated with poor survival in soft tissue sarcoma patients. PSME1 is involved in immunoproteasome assembly for generating tumor antigens presented by MHC class I molecules. In this study, we aimed to validate PSME1 as a prognostic biomarker in an independent and larger series of soft tissue sarcomas by immunohistochemistry.

**Methods:**

Tissue microarrays containing leiomyosarcomas (n = 34), myxofibrosarcomas (n = 14), undifferentiated pleomorphic sarcomas (n = 15), undifferentiated spindle cell sarcomas (n = 4), pleomorphic liposarcomas (n = 4), pleomorphic rhabdomyosarcomas (n = 2), and uterine leiomyomas (n = 7) were analyzed for protein expression of PSME1 using immunohistochemistry. Survival times were compared between high and low expression groups using Kaplan–Meier analysis. Cox regression models as multivariate analysis were performed to evaluate whether the associations were independent of other important clinical covariates.

**Results:**

PSME1 expression was variable among soft tissue sarcomas. In leiomyosarcomas, high expression was associated with overall poor survival (p = 0.034), decreased metastasis-free survival (p = 0.002) and lower event-free survival (p = 0.007). Using multivariate analysis, the association between PSME1 expression and metastasis-free survival was still significant (p = 0.025) and independent of the histological grade.

**Conclusions:**

High expression of PSME1 is associated with poor metastasis-free survival in soft tissue leiomyosarcoma patients, and might be used as an independent prognostic biomarker.

**Electronic supplementary material:**

The online version of this article (doi:10.1186/s13569-016-0057-z) contains supplementary material, which is available to authorized users.

## Background

Soft tissue sarcomas are a heterogeneous group of rare malignancies often having poor outcome [1]. Soft tissue sarcomas constitute less than 1 % of all cancers [1] while there are more than 50 histological subtypes with sometimes overlapping histological features [2]. Distinction is essential as subtypes differ in biological behaviour and sensitivity to chemotherapy, and as such an adequate histological diagnosis, is crucial for clinical decision making [3]. Fifty-six percent of soft tissue sarcomas present as localized disease at the time of diagnosis, and surgery is the mainstay of treatment, sometimes combined with radiotherapy or chemotherapy [4].

From the molecular point of view, soft tissue sarcomas can be distinguished into two categories. The first class includes sarcomas with a simple genome, in which recurrent translocations, amplifications or specific mutations can be found. The second class includes sarcomas with a complex genome, characterized by a multitude of chromosomal alterations and genomic instability, often reflected by pleomorphic histological features [3]. This group includes high grade leiomyosarcoma, myxofibrosarcoma, undifferentiated pleomorphic sarcoma, undifferentiated spindle cell sarcoma, pleomorphic liposarcoma, and pleomorphic rhabdomyosarcoma.

Leiomyosarcomas constitute 5–10 % of all soft tissue sarcomas, displaying smooth-muscle differentiation [1]. Studies showed for leiomyosarcoma that the metastasis-free 5-year survival rate is about 60 % [5]. Histological grade is the most important prognostic factor for most soft tissue sarcomas. By using FNCLCC grading system, which is the most widely used 3-grade system, soft tissue sarcomas are divided into low, intermediate and high grade based on the sum score of three histologic parameters including tumor differentiation, mitotic count and tumor necrosis. About 65 % of leiomyosarcomas are reported to have high-grade areas [6]. High grade leiomyosarcomas often have poor patient outcome [4]. Until now, the genetics and pathology of leiomyosarcomas are not completely understood and as they have a complex genome, no molecular diagnostic tests or specific therapeutic targets are available. Hence, there is a strong need for new molecular markers that can aid in the stratification of leiomyosarcomas patients with respect to their disease outcome.

In a previous study, we used imaging mass spectrometry to compare these soft tissue sarcomas with a complex genome. A panel of protein signatures that could distinguish between different subtypes, or were associated to patient survival were discovered [7]. Among them, proteasome activator complex subunit 1 (PSME1) was found indicative of poor survival in soft tissue sarcomas. PSME1 (also known as REGalpha and PA28A), is a multicatalytic proteinase complex, implicated in immunoproteasome assembly and required for efficient antigen processing [8]. Intriguingly, PSME1 was also found to associate with diagnosis or prognosis in other tumor types, e.g. prostate cancer [9], breast cancer [10] and ovarian cancer [11, 12].

In this study, we used tissue microarrays of soft tissue sarcomas with complex genomes, to evaluate whether PSME1 expression can predict clinical outcome in soft tissue sarcomas, especially leiomyosarcomas.

## Methods

### Tissue microarrays

Tissue microarrays were previously constructed from paraffin embedded formalin fixed tissues using a semi-automated TMA apparatus (TMA Master; 3D Histech, Budapest, Hungary) [13]. Clinicopathological details were described previously [14]. In brief, analysed samples include 34 leiomyosarcomas, 14 myxofibrosarcomas, 15 undifferentiated pleomorphic sarcomas, four undifferentiated spindle cell sarcomas, four pleomorphic liposarcomas, two pleomorphic rhabdomyosarcomas, and seven uterine leiomyomas. Clinicopathological data for the leiomyosarcomas, as described previously [14], are summarized in Additional file 1: Table S1. All tumor samples are present at least in triplicates with a diameter of 1.5 mm (a surface area of around 1.767 mm^2^). Cores from colon, liver, placenta, prostate, skin, and tonsil were included for control and orientation purposes. Four micrometre thick sections were transferred by using a tape-transfer system to coated glass slides for analysis.

The histological diagnosis of all samples was confirmed by reviewing the hematoxylin and eosin—stained slides by expert pathologist (J. V. M. G. B.). Malignant tumors were graded according to the FNCLCC (French Fédération Nationale des Centres de Lutte Contre le Cancer) grading system [1]. All samples were handled according to the Dutch code of proper secondary use of human material as accorded by the Dutch society of pathology (Federa). The samples were handled in a coded manner. All study methods were approved by the LUMC ethical board (B16.025).

### PSME1 immunohistochemistry

Four micrometre thick sections were dried overnight at 37  °C. Immunohistochemistry was performed using anti-PSME1 antibody (clone [EPR10968(B)], abcam, Cambridge, UK) according to protocols described previously [15]. Briefly, slides underwent deparaffinization, blocking of endogenous peroxidase, antigen retrieval (10 min microwave in citrate, pH 6.0), pre-incubation, and addition of the primary antibody in a dilution of 1:1500 overnight. Next, slides were incubated with Poly-HRP-GAM/R/R [Immunologic BV, Duiven, The Netherlands (DPVO110HRP)], visualized with DAB+Substrate Chromogen System (DAKO, Heverlee, Belgium) and counterstained with hematoxylin. Colon tissue was used as a positive control. As a negative control slides were incubated with PBS/1 % BSA instead of the primary antibody.

### Scoring of immunohistochemistry

Slides were scored independently by two observers (J.V.M.G.B and A.H.G.C) as described previously [16]. In brief, staining intensity (0, absent; 1, weak; 2, moderate; 3, strong) and percentage of positive tumor cells (0, 0 %; 1, 1–24 %; 2, 25–49 %; 3, 50–74 %; 4, 75–100 %) were assessed. Afterwards, scores of staining intensity and percentage of positive tumor cells were added to obtain the sum score; for later statistical analysis, the average sum score was calculated over all cores belonging to the same tumor. Proteasomes are present both in the nucleus as well as in the cytoplasm of eukaryotic cells, although their relative abundance within these compartments can be highly variable [8, 17–20]. We therefore evaluated cytoplasmic and nuclear staining separately. Cores in which tissue was lost or with not enough tumor area were excluded from the analysis. Cores with differences on sum score from two observers more than two were re-evaluated to reach consensus.

### Statistical analysis

Only primary tumour samples were used in statistical analysis. First, the distribution of sum score data was evaluated by Shapiro–Wilk normality test. As this test showed that the score data was not normally distributed, nonparametric Spearman correlation coefficient was used as a measure of the statistical dependence between the histological grades and PSME1 expression. Further statistical two-group comparisons between controls (uterine leiomyoma) and the different histological grades of soft tissue sarcomas were calculated by Dunn’s multiple-comparison test. Spearman correlation was performed in R environment (R Foundation for statistical Computing, Vienna, Austria), scatter plots and Dunn’s test results were generated in GraphPad Prism version 6.00 for Windows (GraphPad Software, La Jolla, California, USA, http://www.graphpad.com). All two-sided p values equal or lower than 0.05 were considered statistically significant.

For survival analysis, patients were dichotomized into two groups. We dichotomized leiomyosarcoma patients into high and low expression groups according to the sum scores of immunohistochemistry, for which we chose the cut-off at the 3rd quartile—Experience shows that molecular subgroups are usually found in 10–25 % of the patients (e.g. HER2 overexpression [21], KRAS mutation [22]). Differences in overall survival, metastasis-free survival and event-free survival between these groups were investigated using Kaplan–Meier curves and the log-rank test. Independent variables predicting survival were evaluated in a multivariable model using Cox Regression analyses. Survival analysis was performed in R environment (R Foundation for statistical Computing, Vienna, Austria) using *Survival* package and all two-sided p values lower or equal than 0.05 were considered statistically significant.

## Results

### Variable nuclear and cytoplasmic expression of PSME1 in soft tissue sarcomas

**Fig. 1:**
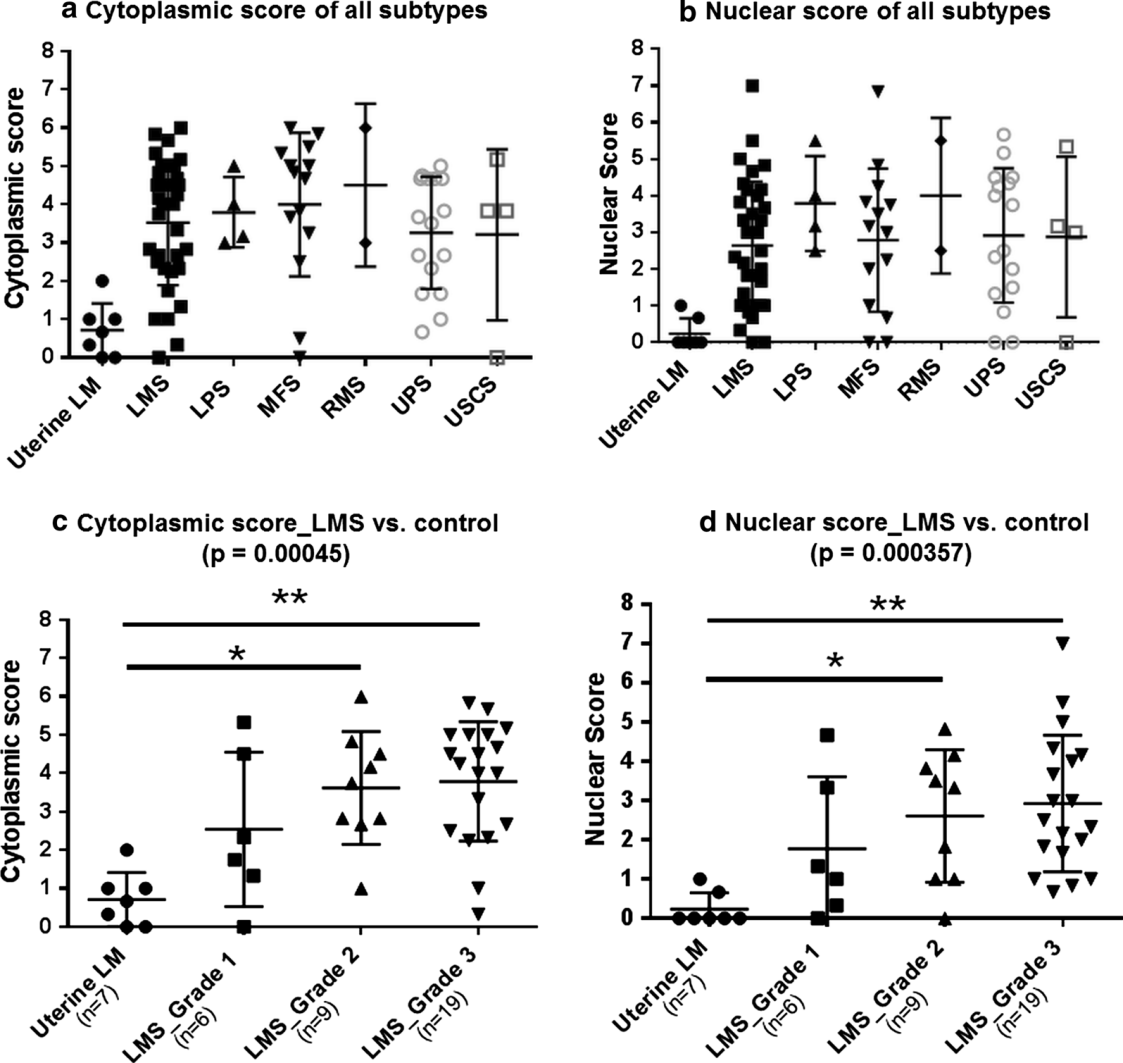
Summary of PSME1 immunohistochemistry results. Variable expression of PSME1 both in the cytoplasm (**a**) as well as in the nucleus (**b**) in soft tissue sarcomas, while expression in uterine leiomyoma (LM; control) is low. *LMS* leiomyosarcomas, *LPS* pleomorphic liposarcomas, *MFS* myxofibrosarcomas, *RMS* pleomorphic rhabdomyosarcomas, *UPS* undifferentiated pleomorphic sarcomas, and *USCS* undifferentiated spindle cell sarcomas. In leiomyosarcomas, both cytoplasmic (**c**) and nuclear (**d**) expression increased with increasing histological grade (p = 0.00045 and p = 0.000357). In addition, both cytoplasmic and nuclear expression of PSME1 was significantly higher in intermediate and high grade leiomyosarcomas as compared to uterine leiomyomas (p ≤ 0.05/p ≤ 0.01). All score data for each group were presented in mean ± SD

**Fig. 2:**
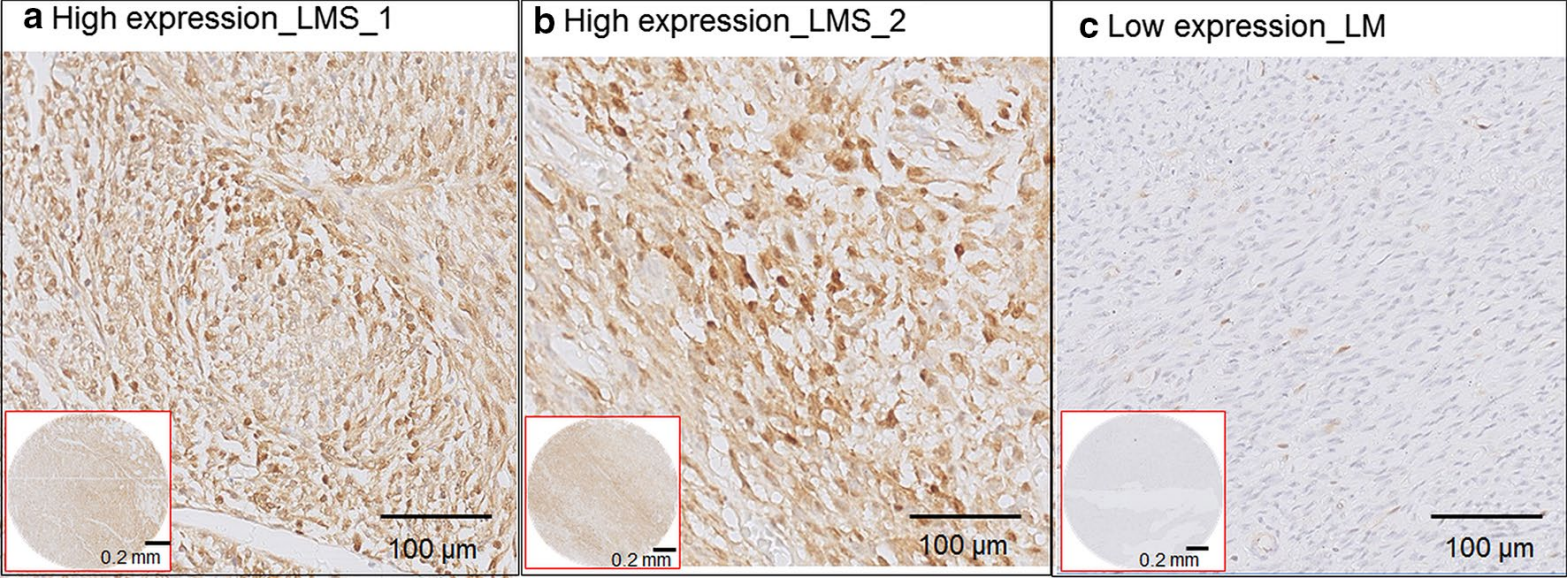
Representative images of immunohistochemistry of PSME1. **a** and **b** are two leiomyosarcoma (LMS) samples with high expression of PSME1. **c** A uterine leiomyoma (LM) control sample with low expression of PSME1. Images in *red squares* are the overviews of expression the tissue microarray cores for respective samples

In soft tissue sarcomas, PSME1 protein expression was found in the majority of the cases, both in the nucleus as well as in the cytoplasm. In contrast, expression in benign leiomyoma was low or absent (Fig. 1a, b). Representative images of immunohistochemistry are shown in Fig. 2.

### Increased expression of PSME1 with increasing histological grade in leiomyosarcomas

The leiomyosarcoma subgroup was large enough to analyse a possible correlation with histological grade. Indeed, while expression was low to absent in uterine leiomyoma, expression gradually increased with increasing histological grade in both nucleus (p_overall_ = 0.000357) and cytoplasm (p_overall_ = 0.00045) in leiomyosarcomas (Fig. 1c, d). Further statistical two groups comparisons between control and any histological grade by Dunn’s multiple comparisons test showed that both nuclear and cytoplasmic staining significantly differed in uterine leiomyomas versus leiomyosarcomas grade 2 (p ≤ 0.05) and uterine leiomyomas versus leiomyosarcomas grade 3 (p ≤ 0.01).

### High nuclear expression of PSME1 predicts poor outcome in leiomyosarcoma patients

**Fig. 3:**
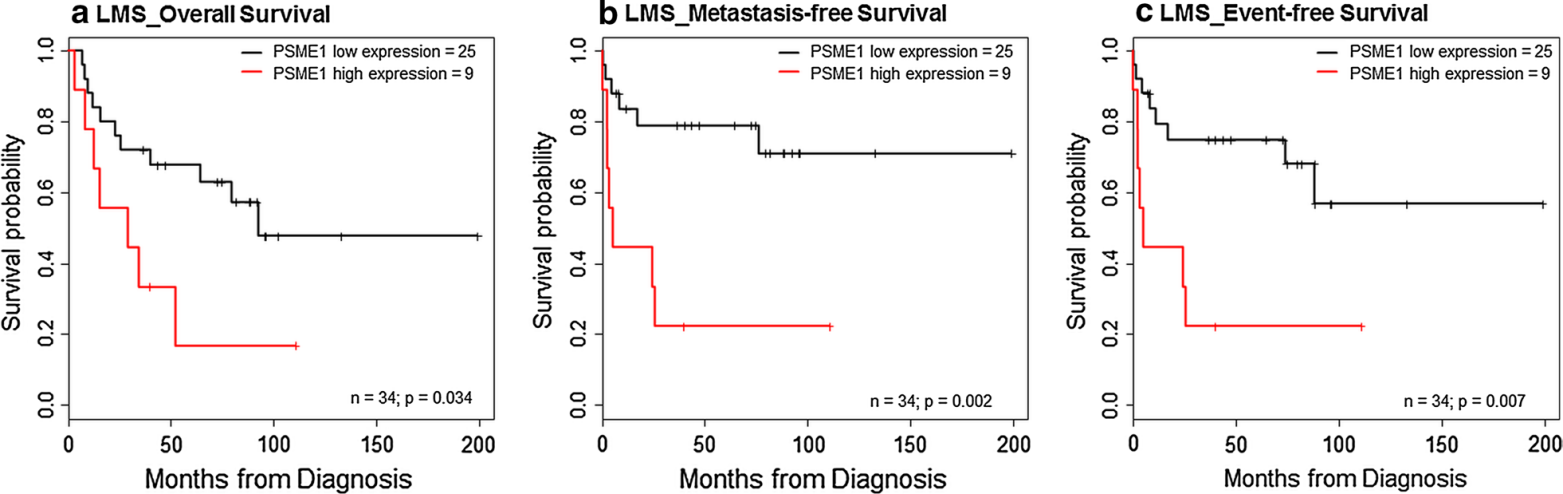
Kaplan–Meier survival plots of PSME1. Kaplan–Meier plots comparing the different survival data of leiomyosarcoma patients with respect to a high and low nuclear expression of PSME1 (cut-off: 3rd quartile). High nuclear expression of PSME1 in leiomyosarcoma was significantly associated with decreased overall survival, metastasis-free survival and event-free survival (log-rank test; p ≤ 0.05)

To investigate a possible correlation of PSME1 expression with clinical outcome, leiomyosarcoma patients were dichotomized into high and low PSME1 expression groups according to the sum scores of immunohistochemistry. High PSME1 expression was associated with poor overall survival (p = 0.034), decreased metastasis-free survival (p = 0.002) and lower event-free survival (p = 0.007) (Fig. 3).

### High nuclear expression of PSME1 as an independent prognostic factor in leiomyosarcoma patients

Using multivariable Cox Regression analyses including clinically relevant co-factors such as histological grade, age and gender, we showed that high nuclear expression of PSME1 was independently associated with metastasis-free survival (p = 0.03) (Table 1). The independent predictive power of nuclear PSME1 expression for overall and event-free survival was at the border of significance (p = 0.07) (Table 1).

**Table 1:** Results of multivariable analysis of factors influencing survival

Clinical association	Variable	Hazards ratio	95 % Confidence interval	*p* value
Metastasis-free survival				
	PSME1 high nuclear expression	3.685	1.177–11.541	0.025*
	Histological grade	1.831	0.710–4.723	0.211
	Age	0.974	0.932–1.017	0.225
	Gender (M)	0.377	0.070–2.039	0.257
Event-free survival				
	PSME1 high nuclear expression	2.667	0.919–7.743	0.071
	Histological grade	2.216	0.882–5.569	0.090
	Age	0.975	0.937–1.015	0.215
	Gender (M)	0.339	0.068–1.695	0.188
Overall survival				
	PSME1 high nuclear expression	2.612	0.916–7.448	0.072
	Histological grade	2.552	0.953–6.837	0.062
	Age	1.005	0.972–1.039	0.758
	Gender (M)	2.071	0.660–6.502	0.212

* *p* value reaches statistically significant level (*p* ≤ 0.05)

## Discussion

Using imaging mass spectrometry we previously identified PSME1 as a prognostic biomarker indicating poor survival in soft tissue sarcoma patients [7]. Imaging mass spectrometry is a sensitive discovery tool (zepto-molar sensitivity [23]) enabling the detection of hundreds of molecules directly from tissue [24, 25]. To further explore the prognostic value of PSME1 we analysed PSME1 expression in a larger, independent set of soft tissue sarcomas using immunohistochemistry on tissue microarrays. PSME1 (or PA28A) encodes a subunit of the proteasome system, which is a major source for generation of tumor antigens presented by MHC class I molecules [26, 27]. Escape of immune response is one of the hallmarks of cancer [28]. In addition, elevated proteasome activity in tumor cells has been described to influence transcription factors involved in cell survival or apoptosis [29, 30]. Novel strategies using the proteasome have been proposed for cancer treatment for example by alternating the NAD^+^/NADH ratio to change kinetics of proteasomal degradation [30] or inhibiting proteasome to induce apoptosis [31–34].

PSME1 is expressed in many different cell types, especially antigen presenting cells, and its expression can be controlled by interferon gamma. Both chemotherapy and TNF-alpha may induce a local inflammatory reaction within the tumor microenvironment and therefore may influence expression of PSME1. It is of interest that all sarcoma subtypes included in our study expressed PSME1 to a variable extent, while neoadjuvant chemotherapy or treatment with interferon gamma is not standard practice in our hospital. As far as clinical data were available, only four patients received preoperative chemotherapy or TNF-alpha, and expression levels were not significantly different. In the control group, consisting of uterine leiomyomas, expression was low to absent, both in the nucleus as well as in the cytoplasm.

High PSME1 expression was also described in other tumors. For example, increased PSME1 expression was also found in primary and metastatic human prostate cancer and was suggested as a potential target for therapeutic intervention [9]. PSME1 was previously also detected using imaging mass spectrometry in other tumors: Dekker et al. detected PSME1 as a marker of stromal activation in breast cancer [10]. Previous studies also showed that PSME1 could be a molecular signature to discriminate between benign and malignant ovarian tumors [11, 35], and an early diagnosis and tumor-relapse biomarker [12]. Zhang et al. detected PSME1 as a tumor marker in human oesophageal squamous cell carcinoma [36]. The proteasome can be present in the cytoplasm as well as in the nucleus of all eukaryotic cells, although their distribution and function can be variable [17]. We here show that in soft tissue sarcomas with a complex genome, PSME1 is expressed both in the cytoplasm and in the nucleus. Proteasome-dependent protein degradation is important in the cytoplasm for MHC class 1 antigen presentation [8]. In the nucleus, PSME1 plays an important role in maintaining the nuclear function including gene expression and cell proliferation [19, 37].

To further evaluate its clinical relevance, we analysed the largest subgroup, comprising 34 leiomyosarcomas of different histological grade, in more detail. Both nuclear as well as cytoplasmic expression of PSME1 significantly increased with increasing histological grade. Moreover, high nuclear expression of PSME1 was significantly associated to poor outcome (overall survival, metastasis-free survival and event-free survival) in leiomyosarcoma patients, although the patient cohort is rather small (n = 34). In multivariate analysis only the association with decreased metastasis-free survival was independent of histological grade, while an independent association to poor overall survival and decreased event-free survival was at the border of significance. Although PMSE1 expression is a promising biomarker, our results need to be validated in an independent cohort of leiomyosarcomas.

In summary, we found elevated expression of the proteasome subunit PSME1 in leiomyosarcomas compared to control tissues, and an association of the expression with increasing histological grade in leiomyosarcoma. Moreover, high nuclear PSME1 expression was found to be an independent predictor of metastasis-free survival in leiomyosarcoma patients. Our results suggest that the expression of proteasome subunits such as PSME1 could be taken into account for leiomyosarcoma patients when considering immunotherapeutic strategies in these tumors [38].

## Conclusions

We show variable expression of PSME1 in different soft tissue sarcoma subtypes with complex genomes. Our results showed that high nuclear expression of proteasome activator complex subunit 1 is an independent poor prognostic factor in leiomyosarcomas, which suggests that the proteasome could be exploited as a possible novel target for the treatment of leiomyosarcomas.

## Additional file

10.1186/s13569-016-0057-z Clinicopathologic characterization of leiomyosarcoma patients [14].

## Abbreviations

PSME1: proteasome activator complex subunit one
LMS: leiomyosarcomas
MFS: myxofibrosarcomas
UPS: undifferentiated pleomorphic sarcomas
Uterine LM: uterine leiomyomas
LPS: pleomorphic liposarcomas
RMS: pleomorphic rhabdomyosarcomas
USCS: undifferentiated spindle cell sarcomas
