# Supplementary material for: High nuclear expression of proteasome activator complex subunit 1 predicts poor survival in soft tissue leiomyosarcomas
Source: Clin Sarcoma Res. 2016 Oct 1;6:17. doi: 10.1186/s13569-016-0057-z (PMC5045577; doi:10.1186/s13569-016-0057-z)
Supplement: Supplementary file 1 — 10.1186/s13569-016-0057-z Clinicopathologic characterization of leiomyosarcoma patients [14]. [file 13569_2016_57_MOESM1_ESM.docx]

Supplementary information

Additional Table S1.

| Feature |  | Value |
| --- | --- | --- |
| No. patients |  | 34 |
| Age | Median (years) | 63 |
|  | Range (years) | 30-86 |
| Gender | Male : Female | 13: 21 |
| Histological grade | Grade 1: Grade 2: Grade 3 | 6: 9: 19 |
| Length of follow-up | Median metastasis-free survival (months) | 56.9% at max.follow-up length |
|  | Median event-free survival (months) | 87.2 |
|  | Median overall survival (months) | 80.2 |
